# Evolution of the Population Structure of *Staphylococcus pseudintermedius* in France

**DOI:** 10.3389/fmicb.2018.03055

**Published:** 2018-12-13

**Authors:** Marine Bergot, Patricia Martins-Simoes, Hélène Kilian, Pierre Châtre, Kate A. Worthing, Jacqueline M. Norris, Jean-Yves Madec, Frédéric Laurent, Marisa Haenni

**Affiliations:** ^1^Centre International de Recherche en Infectiologie (CIRI), Equipe Pathogénie des Staphylocoques, Faculté de Médecine Laennec, Lyon, France; ^2^Institut des Agents Infectieux – Centre de Biologie Nord, Centre National de Référence des Staphylocoques, Hospices Civils de Lyon, Lyon, France; ^3^Unité Antibiorésistance et Virulence Bactériennes, Université de Lyon – Agence Nationale de Sécurité Sanitaire (Anses), Lyon, France; ^4^Sydney School of Veterinary Science, University of Sydney, Camden, NSW, Australia; ^5^Department of Microbiology and Immunology, Peter Doherty Institute for Infection and Immunity, The University of Melbourne, Parkville, VIC, Australia

**Keywords:** dog, MRSP, MLST, ST258, ST496

## Abstract

*Staphylococcus pseudintermedius* is a colonizer as well as an important pathogen of dogs where it is responsible for skin, ear and post-operative infections. The emergence of methicillin-resistant *S. pseudintermedius* (MRSP) in the early 2000s, which were additionally resistant to most veterinary-licensed antibiotics, drew specific attention to these pathogens due to the limitations created in veterinary therapeutic options. Multiple studies showed that the sequence type (ST)71 was the most frequently identified clone in Europe. A few years ago, several publications have suggested a decline of the ST71 clone and the emergence of the ST258 lineage in Northern Europe. In this study, we show that ST71 is also decreasing over time in France and that the non-ST71 population is highly heterogeneous. Globally, the non-ST71 clones are more susceptible to antibiotics, which might be good news for veterinarians. Two other lineages, ST258 and ST496, seem to be successful in France. These isolates, as well as representatives of the ST71 clone, underwent whole-genome sequence. This study shows that the ST71 and ST496 clusters are highly homogenous while the ST258 cluster is more diverse. Each ST possesses a specific pattern of resistance and virulence genes. The reasons for the apparent and simultaneous success of the ST258 and ST496 clones remain unclear. But the emergence of the ST496 clone will require monitoring given its multi-resistant genotype and threat to canine health.

## Introduction

*Staphylococcus pseudintermedius* (SP), initially described in 2005, is a normal commensal bacterium in dogs. However, it is also recognized as the most frequent opportunistic pathogen responsible for skin, ear and surgical site infections and complications in dogs. While its natural hosts are dogs, human infections typically associated with exposure to dogs have been increasingly reported in the last few years (Lozano et al., 2017; Pomba et al., 2017), highlighting the zoonotic potential of *S. pseudintermedius* (Somayaji et al., 2016).

Similar to *S. aureus*, methicillin-resistance in *S. pseudintermedius* has been recognized as a major issue in veterinary medicine. Since the first description of methicillin-resistance *S. pseudintermedius* (MRSP) in 1999 (Gortel et al., 1999), its global distribution has expanded and the number of clinical infections associated with MRSP in companion animals (mostly dogs) has dramatically increased. With the reported prevalence as high as 60% (Kawakami et al., 2010; Haenni et al., 2014; Worthing et al., 2018), the situation is worsened by the high frequency of multi and extensively drug resistant strains markedly restricting the therapeutic arsenal for veterinarians (Perreten et al., 2010).

Methicillin-resistance *S. pseudintermedius* has been spreading worldwide through the dissemination of specific lineages from different genetic backgrounds. The most prevalent clones are ST71 in Europe, ST68 in the United States and ST45/ST112 in Asia (Pires Dos Santos et al., 2016). However, several studies in Northern countries of Europe showed that ST71 MRSP may be challenged by other less frequent clones such as ST258 MRSP (Kjellman et al., 2015; Damborg et al., 2016; Duim et al., 2016; Gronthal et al., 2017). In 2010, we detected non-ST71 MRSP lineages in French veterinarian isolates, including two ST258 isolates (Haenni et al., 2014). This ST258 MRSP lineage was reported to be more frequently susceptible than the ST71 MRSP to enrofloxacin, gentamicin, and chloramphenicol, while also being marginally more resistant to trimethoprim-sulfonamides and tetracyclines (Pires Dos Santos et al., 2016). The same trend toward susceptibility to veterinary-licensed antibiotics has also been observed for other lineages, so that the emergence of a polyclonal non-ST71 MRSP population may be good news for the treatment of such infections (Haenni et al., 2014; Kjellman et al., 2015). Moreover, non-ST71 MRSP lineages also should be less virulent as they form less biofilm and show lower adherence to canine corneocytes (Osland et al., 2012; Latronico et al., 2014).

In France, the occurrence of MRSP in clinical samples from dogs is continuously monitored through Resapath, the French network for surveillance of antimicrobial resistance in clinical pathogens. We utilized this network to compare two collections (2012–2013 and 2015–2016) of MRSP isolates collected from canine skin and soft tissue infections. The aims of our study were firstly to assess if a shift in the population structure of MRSP, from ST71 toward non-ST71 lineages, has occurred in France, and secondly to characterize the resistome and virulome of the most prevalent clones by using whole genome sequencing.

## Materials and Methods

### Bacterial Sampling and Identification

Methicillin-resistance *S. pseudintermedius* isolated from skin and soft tissue infections in dogs were collected from the veterinary laboratories of Resapath, the network for surveillance of antimicrobial resistance in clinical pathogens in France^1^. Two collections were constituted: the first one presented 95 isolates sampled in 2012–2013, and the second one 87 isolates collected in 2015–2016. Each clinical isolate corresponded to a unique animal. All isolates were sent to the Anses laboratory in Lyon, France, for further analysis. Species identification was performed by PCR-RFLP using *Taq*I restriction analysis on the *kat* gene (Blaiotta et al., 2010). The presence of the *mecA* and 16S rRNA genes were detected by PCR (Maes et al., 2002) and used to confirm methicillin resistance and as an internal quality control, respectively.

### Antimicrobial Susceptibility Tests

All *S. pseudintermedius* isolates were tested for their antimicrobial susceptibility by disk diffusion method according to the guidelines of the French Society for Microbiology (CA-SFM ^2^). Antibiotics tested were cefovecin 30 μg (which was used as the phenotypic marker of methicillin resistance), penicillin 6 μg, fusidic acid 10 μg, kanamycin 30 μg, gentamicin 15 μg, tobramycin 10 μg, erythromycin 15 μg, spiramycin 100 μg, lincomycin 15 μg, tetracycline 30 μg, chloramphenicol 30 μg, florfenicol 30 μg, enrofloxacin 5 μg, vancomycin 30 μg, and teicoplanin 30 μg (Mast Diagnostics, Amiens, France). Bacteria were classified as susceptible, intermediate or resistant according to the clinical breakpoints approved by the veterinary part of the CA-SFM. *Staphylococcus aureus* ATCC 25923 was used as the quality control strain.

### Multi-Locus Sequence Typing (MLST) and Clonal Complexes (CC)

Multi-locus sequence typing was performed for all MRSP isolates according to the 7-loci scheme (Solyman et al., 2013). STs were determined using the MLST website^3^ and new STs were assigned by the curator Vincent Perreten (vincent.perreten@vbi.unibe.ch). In addition, for each collection (2012–2013 and 2015–2016), STs were assigned to CCs by Phyloviz v 2.0 (Nascimento et al., 2017), using goeBURST (Francisco et al., 2009) algorithm with default parameters. A clonal complex is composed by all strains sharing 5 or more identical profile alleles (double-loci variants – DVL parameter).

### Whole Genome Sequencing and Analysis

Whole genome sequencing (WGS) was performed on a subset of 23 isolates belonging to the three most prevalent clones in both surveillance studies: all ST258 (*n* = 6), ST496 (*n* = 9), and a subset of ST71 (*n* = 8, among the 110 ST71 identified) were whole-genome sequenced. WGS was performed on genomic DNA extracted using the Microbial DNA extraction kit (Macherey Nagel, Hoerdt, France). The genomic library was prepared using the Nextera XT DNA Library Preparation kit (Illumina Inc., Cambridge, United Kingdom), according to the manufacturer’s protocol, and 2x 250-bp paired-end reads were generated with Illumina sequencing technology (San Diego, CA, United States).

### Assembly and Annotation

We used the Nullarbor pipeline (T. Seemann^4^) to perform paired-end reads trimming using Trimmomatic v 0.33 (Bolger et al., 2014), and the cleaned reads were then assembled using SPAdes v 3.5 (Bankevich et al., 2012). Quality of genome assemblies was assessed with QUAST v 4.6.3 (Mikheenko et al., 2016). Always in the Nullarbor pipeline, the genomes annotation was then performed using Prokka v 1.10 (Seemann, 2014).

### *In silico* MLST

All publicly available genomes (NCBI genomes database, accession date 14.02.2018) *S. pseudintermedius* (*n* = 19) were *in silico* MLST typed using the MLST pipeline^5^.

### Resistome and Virulome

The resistome and virulome of all sequenced ST71, ST258, and ST496 genomes were characterized using the ABRicate v 0.8.1 software (Seeman T^6^) against the ResFinder database as updated on February 20, 2018, and VFDB database (Chen et al., 2005; Zankari et al., 2012). Two curated in-house databases (DB) were subsequently built and the genomes were screened against these databases, using ABRicate v 0.8.1, to search for homologous genes. As resistance genes of *S. pseudintermedius* are poorly described, and because *S. aureus* share common resistance genes with *S. pseudintermedius*, we built the first database (DB_SA) using 336 markers used in the *S. aureus* DNA microarray Genotyping kit v2 (Monecke et al., 2008). Furthermore, we performed a state of the art of reported virulence-associated genes in *S. pseudintermedius* and built the second database (DB_SP) using a total of 49 genes : *spsA-R, clpP, siet, speta, se-int, lukF-I, lukS-I, sec-canine, exi, expB, agrA-D, icaA-D, nanB, coa, clpX, saeR, saeS, htrA, nucC, hlb, sigB, srrA, sarA, rot, traP, expA*, and *epbS* (Ben Zakour et al., 2011; Bannoehr et al., 2012; Garbacz et al., 2013; Pietrocola et al., 2015; Couto et al., 2016; Ruzauskas et al., 2016). All gene sequences used in DB_SP, except the *sec-canine* gene, were obtained from the complete genome of *S. pseudintermedius* strain ED99 (GenBank accession number: CP002478.1).

Because *sec*-*canine* gene was not available for *S. pseudintermedius*, we used the *sec*-*canine* gene of the closest phylogenetic species *S*. *intermedius* (strain 95-011195, GenBank accession number: U91526.1) and we included it in DB_SP.

Presence of both virulence and resistance-associated genes was initially detected: (i) if ≥50% of the gene length and ≥50% nucleotide identity were detected when using the DB_SA; (ii) if ≥90% of the gene length and ≥70% nucleotide identity were detected when using the DB_SP. For both databases (DB_SP and DB_SA), we used ABRicate to identify the presence of virulence and resistance-associated genes. As DB_SA was built based on *S. aureus* genes, we used a lower identity threshold (i.e., 50%) than the one used for DB_SP (i.e., 90%) to be able to detect homologous genes. Then, for the DB_SA, a second validation of positive hits was performed manually using a threshold of at least 80% of the query sequence length. We also screened for the presence of putative CRISPR regions, using CRISPRcasfinder (Grissa et al., 2007).

### Fluoroquinolone Resistance

Mutations in topoisomerase II (*gyrA*) and IV (*grlA*) are known to confer fluoroquinolone resistance in S. *pseudintermedius* (Descloux et al., 2008). Detection of mutations Ser84Leu in *gyrA* and Ser80Ile in *grlA* sequences was performed using Snippy v 3.1^6^. The fluoroquinolone-susceptible *S. pseudintermedius* strain CCUG49543^T^ (also known as LMG 22219^T^) *gyrA* and *grlA* sequences as reference (accession numbers AM262968 and AM262971, respectively) (Devriese et al., 2005). Visual confirmation of non-synonymous mutations was performed with Seaview (Gouy et al., 2010).

### SNP Based Phylogeny

SNP detection was performed with Snippy v.3^7^ to construct a SNP based phylogeny for ST71, ST258, and ST496. Strain ED99 (ST25) was used as a reference outgroup/external ST to root the tree. A raw phylogenetic tree using the approximately-maximum-likelihood (ML) model and generalized time-reversible (GTR) models of nucleotide evolution were built using FastTree2 (Price et al., 2010). Strain ED99 was used to root the phylogenetic tree. To visualize and annotate the trees we used the bioconductor package *ggtree* (Yu et al., 2017). In addition, a pairwise SNP distance matrix was built with snp-dists v 0.6^8^, as implemented in Snippy v.3.

### Statistical Analysis

Proportions of antimicrobial resistance in ST71 isolates vs. non-ST71 isolates were compared using the chi-squared test. Differences were considered significant if *P* < 0.05.

### Ethics Statement

No ethical approval was needed since this study did not involve any experimentation on animals. Only bacteria collected from clinical samples were used in the frame of this study.

### Data Availability

The data sets supporting the results of this article are available from the Sequence Read Archive (SRA) under the BioProject no. PRJNA498009.

## Results

### Population Structure of French MRSP

ST71 MRSP remains the dominant methicillin-resistant clone in France (Table 1) but its prevalence over the years revealed a decreasing trend, even not statistically significant (*p* = 0.1643), from 65.3% in the 2012–2013 collection to 55.2% in the 2015–2016 collection. The non-ST71 MRSP group is largely polyclonal, with most STs occurring only once. Moreover, seventeen STs were newly identified and assigned to the following numbers: 406, 407, 408, 410, 413, 414, 415, 416, 419, 420, 421, 424, 425, 1026, 1027, 1028, and 1029. The ST258 MRSP lineage was detected in one isolate in 2012–2013 and in five isolates in 2015–2016, which were all isolated in different counties. Conversely, ST261 was identified in eight isolates from eight different counties in 2012–2013 but only in one isolate in 2015–2016. Finally, we observed the emergence of the ST496 MRSP clone, which was absent in 2012–2013 and was found in nine isolates from four different counties in 2015–2016.

**Table 1:** Distribution of the STs per collection.

Sequence type (ST)	No. of isolates (%)	
	2012–2013 (*n* = 95)	2015–2016 (*n* = 87)
71	62 (65.3)	48 (55.2)
45	2 (2.1)	0
68	1 (1.1)	0
85	1 (1.1)	3 (3.4)
136	0	1 (1.1)
181	1 (1.1)	1 (1.1)
196	1 (1.1)	0
258	1 (1.1)	5 (5.7)
261	8 (8.4)	3 (3.4)
294	2 (2.1)	2 (2.3)
298	0	1 (1.1)
339	0	1 (1.1)
342	0	2 (2.3)
406	1 (1.1)	0
407	1 (1.1)	0
408	1 (1.1)	0
410	1 (1.1)	0
413	1 (1.1)	0
414	4 (4.2)	1 (1.1)
415	1 (1.1)	0
416	1 (1.1)	0
419	1 (1.1)	0
420	1 (1.1)	0
421	1 (1.1)	0
424	1 (1.1)	0
425	1 (1.1)	0
450	0	1 (1.1)
496	0	9 (10.3)
551	0	1 (1.1)
1026	0	1 (1.1)
1027	0	1 (1.1)
1028	0	1 (1.1)
1029	0	1 (1.1)

### Antibiotic Susceptibility Profiles

ST71 MRSP was highly resistant to veterinary-licensed antibiotics, with chloramphenicol, tetracyclines, and fusidic acid being the last antibiotics with resistance rates lower than 90% in 2015–2016 (Tables 2, 3). Between 2012–2013 and 2015–2016, we observed a significant increase in the resistance to tobramycin, kanamycin, and chloramphenicol. In non-ST71 MRSP, we observed a higher rate of resistance to gentamicin (97.4% in 2015–2016), macrolides-lincosamides (84.8 and 63.6%, respectively, due to the presence of the inducible MLS_B_ phenotype). Compared to ST71 MRSP isolates, the rate of resistance to kanamycin, tobramycin, and enrofloxacin (30.3% vs. >90%) was lower in non-ST71 MRSP but higher for tetracyclines (78.8% vs. 37.1%).

**Table 2:** Antimicrobial resistance in isolates collected in 2012–2013 (*n* = 95).

Antibiotic	Breakpoints (mm)^1^ R < /S ≥	ST71 (*n* = 62)		non-ST71 (*n* = 33)		*p*
		No. of strains	Percentage	No. of strains	Percentage	
Kanamycin	15/17	62	100.0	29	87.9	0.023
Gentamicin	20/20	57	91.9	10	30.3	<2.10–6
Tobramycin	20/20	59	95.2	10	30.3	<2.10–6
Chloramphenicol	19/22	11	17.7	7	21.2	0.681
Florfenicol	19/22	0	0.0	0	0.0	–
Tetracyclines	17/19	23	37.1	26	78.8	0.0001
Erythromycin	17/22	59	95.2	28	84.8	0.182
Spiramycin	20/20	59	95.2	28	84.8	0.182
Lincomycin	17/21	58	93.5	21	63.6	0.0002
Fusidic acid	24/24	2	3.2	1	3.0	0.573
Enrofloxacin	17/22	61	98.4	10	30.3	<2.10–6
Teicoplanin	17/17	0	0.0	0	0.0	–
Vancomycin	17/17	0	0.0	0	0.0	-

*^*1*^Antibiograms were performed by disk diffusion according to the recommendations of the French CA-SFM.*

**Table 3:** Antimicrobial resistance in isolates collected in 2015–2016 (*n* = 87).

Antibiotic	Breakpoints (mm)^1^ R < /S ≥	ST71 (*n* = 48)		non-ST71 (*n* = 39)		*p*
		No. of strains	Percentage	No. of strains	Percentage	
Kanamycin	15/17	48	100.0	38	97.4	0.44
Gentamicin	20/20	39	81.3	16	41.0	0.0001
Tobramycin	20/20	42	87.5	17	43.6	0.00001
Chloramphenicol	19/22	2	4.2	17	43.6	0.00001
Florfenicol	19/22	0	0.0	0	0.0	–
Tetracyclines	17/19	16	33.3	35	89.7	<0,00001
Erythromycin	17/22	46	95.8	33	84.6	0.15
Spiramycin	20/20	46	95.8	33	84.6	0.15
Lincomycin	17/21	46	95.8	28	71.8	0.0018
Fusidic acid	24/24	1	2.1	3	7.7	0.467
Enrofloxacin	17/22	47	97.9	19	48.7	<0,00001
Teicoplanin	17/17	0	0.0	0	0.0	–
Vancomycin	17/17	0	0.0	0	0.0	–

*^*1*^Antibiograms were performed by disk diffusion according to the recommendations of the French CA-SFM.*

### Phylogeny of French Isolates

**FIGURE 1:**
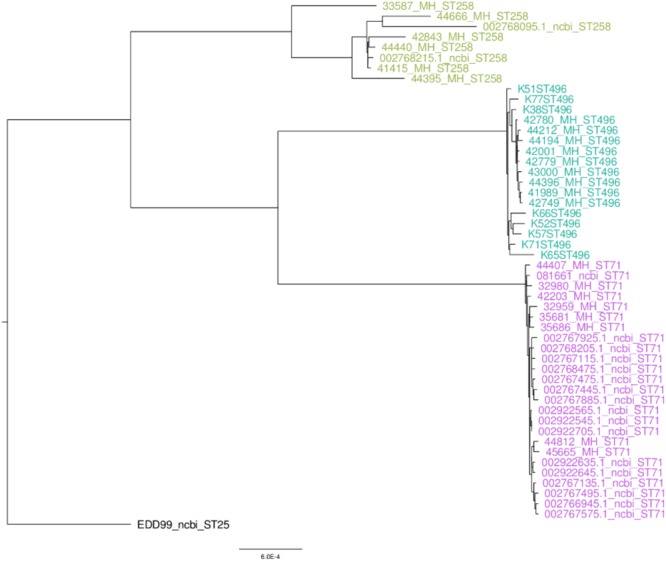
SNP based phylogeny of methicillin-resistant *Staphylococcus pseudintermedius*.

Whole genome sequencing was performed on all ST258 and ST496, as well as on a subset of eight ST71 selected from the two collections (four isolates from each collection) and originating from eight different districts. The phylogenetic tree of the French strains and the ones available on public databases shows that the ST71 and ST496 clades are highly homogenous (Figure 1). On the contrary, the ST258 cluster is more diverse. To support these observations, we used the pairwise SNP distance matrix (Supplementary Table S1) which showed that ST71 is a highly homogenous cluster with an average of 149 SNPs, while ST258 is much more heterogeneous with an average of 1919 SNPs, about thirteen time more than ST71, and twice more than ST496.

### Distribution of Resistance Genes According to MLST Types

**FIGURE 2:**
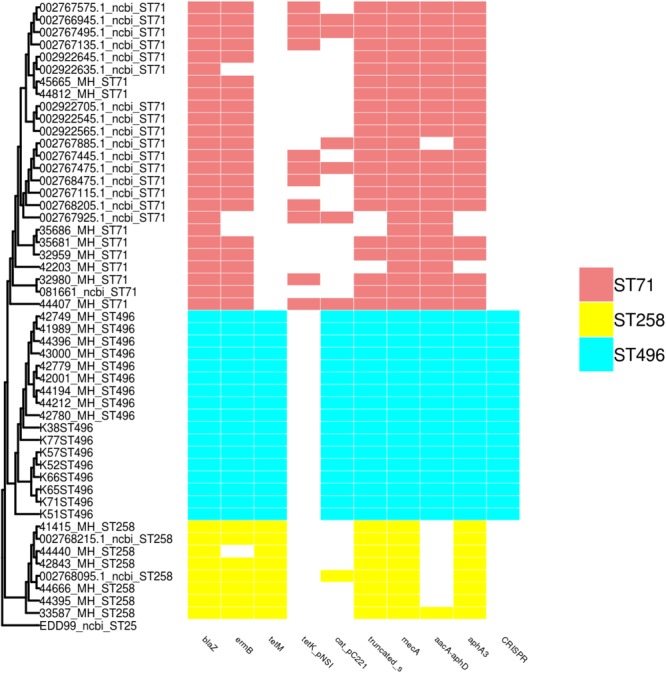
SNP based phylogeny of ST71, ST258, and ST496 with a matrix of resistance associated genes and CRISPR. The SNP based phylogeny was constructed using 19008 SNP. The tip label represents the strain name followed by the ST number. The presence or absence of resistance genes and the CRISPR region are shown as an array of colored boxes on the right: ST71, red boxes; ST258, yellow boxes; and ST496, blue boxes.

Multiple resistance genes were screened (reported in Figure 2), which perfectly correlated with the phenotypic characterization. Each ST presented a specific pattern. For ST496, all isolates displayed an identical pattern, with a higher number of resistance genes when compared to ST71 and ST258. Concerning the ST258 MRSP group, isolates presented a conserved pattern of resistance genes (*blaZ, tet*(M), *aphA3*, truncated *sat* genes) with sporadic variations, while ST71 was more heterogeneous. ST258 and ST496 displayed the *tet*(M) gene, whereas ST71 (when present) displayed the *tet*(K) gene. ST258 only rarely displayed the *aacA*-*aphD* gene conferring resistance to gentamicin, kanamycin, and tobramycin. All ST496 and ST71 isolates were resistant to fluoroquinolones, and they all presented the prototypical mutations Ser84Leu and Ser80Ile in the *gyrA* and *grlA* genes, respectively. On the contrary, all ST258 isolates were susceptible and all isolates presented the intact *gyrA* and *grlA* genes, except one isolate presenting the mutated *grlA* gene.

### Detection of the CRISPR Sequences

CRIPSR sequences were detected in all isolates belonging to the ST496, but were absent in ST71 and ST258 isolates. Typing of the CRISPR elements found in ST496 isolates revealed that they all harbored the same subtype of CRISPR: the subtype IIIA, which is located within the SCC*mec* element found in this lineage.

### Distribution of Virulence Genes According to MLST Types

**FIGURE 3:**
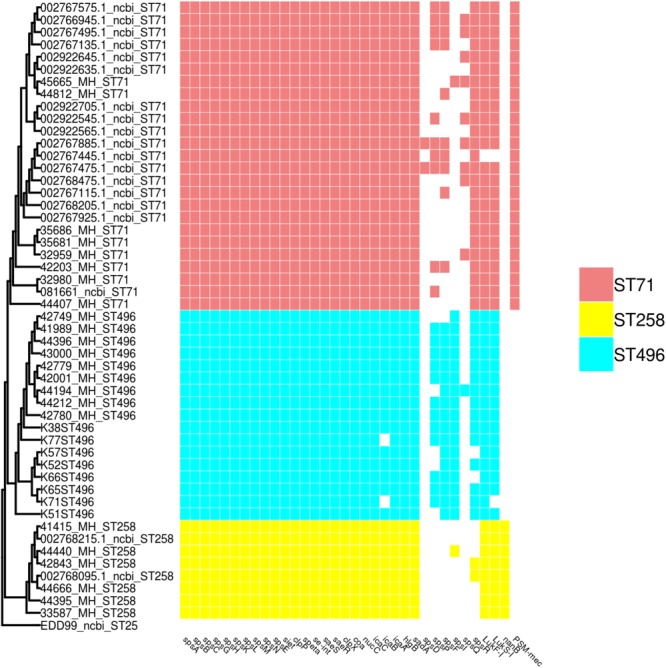
SNP based phylogeny of ST71, ST258 and ST496 with a matrix of virulence associated genes. The SNP based phylogeny was constructed using 19008 SNP. The tip label represents the strain name followed by the ST number. The presence or absence of virulence genes are shown as an array of colored boxes on the right: ST71, red boxes; ST258, yellow boxes; and ST496, blue boxes.

All MLST types shared a core virulence profile, with a group of 24 genes (including the biofilm-associated *ica* operon – except for two ST496 isolates in which *icaB* was absent – and *sps* surface proteins) being systematically present in isolates of all three STs (Figure 3). ST496 isolates were characterized by the presence of the *spsI* gene. ST258 isolates were devoid of the *spsR* gene, but were the only ones to present the *nanB* gene coding for a potential sialidase toxin.

## Discussion

### Decreasing Trend of the ST71 MRSP Lineage

In this study we showed a decreasing trend of ST71 MRSP, with a proportion falling from 65.3% in 2012–2013 to 55.2% in 2015–2016, among the French MRSP associated with canine SSTIs. This clear trend is nevertheless not statistically significant (*p* = 0.1643), due to the number of isolates included, but also mainly to the temporal proximity of the observed populations. A previous French study reported that the proportion of ST71 isolates within MRSP was as high as 82.9% in 2010 (Haenni et al., 2014). The decrease in the ST71 clone between the 2010 study and the 2015–2016 collection is statistically significant (*p* = 0.0023), even though the number of isolates in the 2010 study was low (*n* = 41). Of note, the sampling scheme was not identical since all pathologies were included in the first surveillance study; however, SSTIs represented 63.4% of the reported infections. Taken together, our results corroborate the downward trend of ST71 isolates within the French population of MRSP over the last decade. The decrease of the ST71 MRSP lineage, which has already been reported in several countries from Northern Europe (Duim et al., 2016; Gronthal et al., 2017), thus most likely reflects of a global evolution of the population structure of MRSP in Europe. The number of isolates included in the present study may be considered a limitation. However, all MRSP isolates collected from SSTIs over the two periods were included and the same sampling effort was made in both periods. Considering the robustness of the collection and the number of other studies reporting the same trend in Northern countries, the tendencies detected here can be considered reliable in France.

### Population Structure of Non-ST71 Isolates

In both the 2012–2013 and 2015–2016 collections, as many as 21 distinct non-ST71 STs were identified, the vast majority being found only once. Analysis of the MRSP population structure based on the clonal complexes (CC) represented in our sampling revealed 2 different CCs and 12 singletons in the 2012–2013 collection (Supplementary Figure S1), and 1 CC and 14 singletons in the 2015–2016 collection (Supplementary Figure S2). The presence of many singletons and only a few CCs points out the coexistence of distinct and diverse MRSP population lineages. Identical non-ST71 lineages were rarely identified in both collections. The exceptions are: (i) ST265, which was identified eight times in 2012–2013 and three times in 2015–2016 and thus seems to be on a decreasing trend; (ii) ST258, which was identified once in 2012–2013 and five times in 2015–2016 and, thus, seems to be expanding. Interestingly, a concomitant decrease of ST71 MRSP with the emergence of ST258 MRSP has been previously observed in Finland, Denmark, and Netherlands (Damborg et al., 2016; Duim et al., 2016; Gronthal et al., 2017).

### Clones and Antimicrobial Resistance

Our data show that, despite a higher tetracycline resistance, French ST258 MRSP isolates present a higher susceptibility to gentamicin and sulfonamides (absence of the *aacA*-*aphD* and *cat* genes), but also to fluoroquinolones. These results are consistent with the potential dissemination of a ST258 MRSP in Europe, which may be good news for veterinarians since this lineage is more susceptible than ST71 MRSP to veterinary-licensed antibiotics (Damborg et al., 2016; Duim et al., 2016; Gronthal et al., 2017). The SNP based phylogeny of the French ST71, ST258, ST496 and publicly available ST71, ST258, and ST496 genomes shows a very low diversity within the ST71 and ST496 populations and a more heterogeneous ST258 population. The success of this newly emerging ST258 MRSP lineage remains unknown and cannot be explained by a difference in virulence factors since only *nanB*, a putative sialidase toxin, was identified to be exclusively present in the ST258 isolates. However, as only a few virulence factors have been reported and validated in *S. pseudintermedius*, one cannot exclude the presence or absence of other, yet undescribed, virulence associated genes within this lineage.

### Emergence of the ST496 Clone

In parallel with the emergence of a ST258 MRSP lineage, we also observed the emergence of a novel ST496 MRSP lineage in France in the collection of 2015–2016. All isolates belonging to this new clone were systematically resistant to all veterinary-licensed antibiotics. Of note, over the 87 isolates from the 2015–2016 collection, only the nine ST496 and the three ST85 isolates presented such a large multi-resistance phenotype, with susceptibility present only for florfenicol and fusidic acid. Interestingly, this clone has very recently been reported in Australia (Worthing et al., 2018), where it is described as one of the major MRSP clones, especially in Sydney. Whole-genome comparison of French and Australian isolates shows a very low level of population diversity within this clade, the same resistome and a very close virulome. Compared to isolates of the other two most prominent MRSP lineages in France – ST71 and ST258 – ST496 isolates were the only ones that systematically carried cell wall associated (CWA) genes *spsI* and, except for one isolate, *spsF* genes, which are hypothesized to play a role in pathogenesis as they code for proteins capable of adhesion to extracellular matrix (Bannoehr et al., 2011).

## Conclusion

Put together, our data shows a shift, over the last 6 years, within the population structure of MRSP in France. An apparent decline of the ST71 MRSP lineage is concomitant with the emergence of two novel MRSP lineages in France: the ST258 MRSP lineage from Northern Europe, and a ST496 MRSP lineage which has become the main MRSP clone in Sydney, Australia. Based on the structure of the phylogenetic tree, the ST496 isolates from Australia appear to be ancestral to the French isolates. This is also indicated by the fact that ST496 emerged in Australia in 2013, but was not found in France at that time. The emergence of the ST258 and ST496 lineages in France might be associated with the distribution and importation of animals from other countries. The reasons for their apparent and simultaneous success remain unclear. For ST496, this could be associated with an increased colonization capacity, via the presence of *spsI* and *spsF* genes, but the expression and role of these putative microbial surface components recognizing adhesive matrix molecules (MSCRAMMs) remains to be validated *in vivo*. For ST258, no known virulence factors were exclusively found in this lineage which might help elucidate its success over the ST71 MRSP lineage.

While the decrease in the prevalence of ST71 MRSP is obvious in Europe, it is unclear which other MRSP clone(s) – and for which reasons – will successfully arise. This will have to be closely monitored since the emergence and dissemination of the ST496 MRSP would be a threat to animal health due to its concerning level of antimicrobial resistance.

## Author Contributions

MH, MB, and PM-S designed the experiments and drafted the manuscript. HK and PC performed the laboratory experiments. MB and PM-S performed the genomic analysis. J-YM, FL, KW, and JN actively contributed to the manuscript’s writing. All authors approved the final version of this manuscript.

## Conflict of Interest Statement

The authors declare that the research was conducted in the absence of any commercial or financial relationships that could be construed as a potential conflict of interest.

## Footnotes

1 https://www.resapath.anses.fr/

2 www.sfm-microbiologie.org

3 http://pubmlst.org/spseudintermedius

4 https://github.com/tseemann/nullarbor

5 https://github.com/tseemann/mlst

6 https://github.com/tseemann

7 https://github.com/tseemann/snippy

8 https://github.com/tseemann/snp-dists
