# Supplementary material for: Evolution of the Population Structure of Staphylococcus pseudintermedius in France
Source: Front Microbiol. 2018 Dec 13;9:3055. doi: 10.3389/fmicb.2018.03055 (PMC6300469; doi:10.3389/fmicb.2018.03055)
Supplement: Supplementary file 1 [file Data_Sheet_1.PDF]

	ST71	ST258	ST496
ST71	150	10173	8271
ST258		1919	10404
ST496			832

Table S1:

Mean pairwise SNP distance matrix for ST71, ST496 and ST258, built from SNP alignment with Snippy v 3.1 (https://github.com/tseemann).

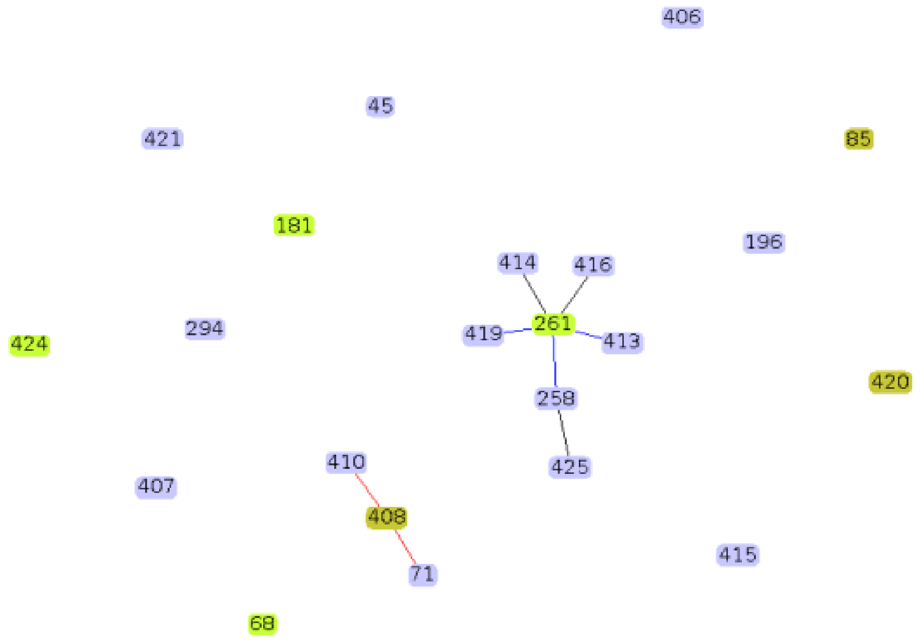

Figure S1:

Clonal Complexes (CCs) distribution for the 2012-2013 collection. Group founders are shown in light green and subgroup founders in dark green.

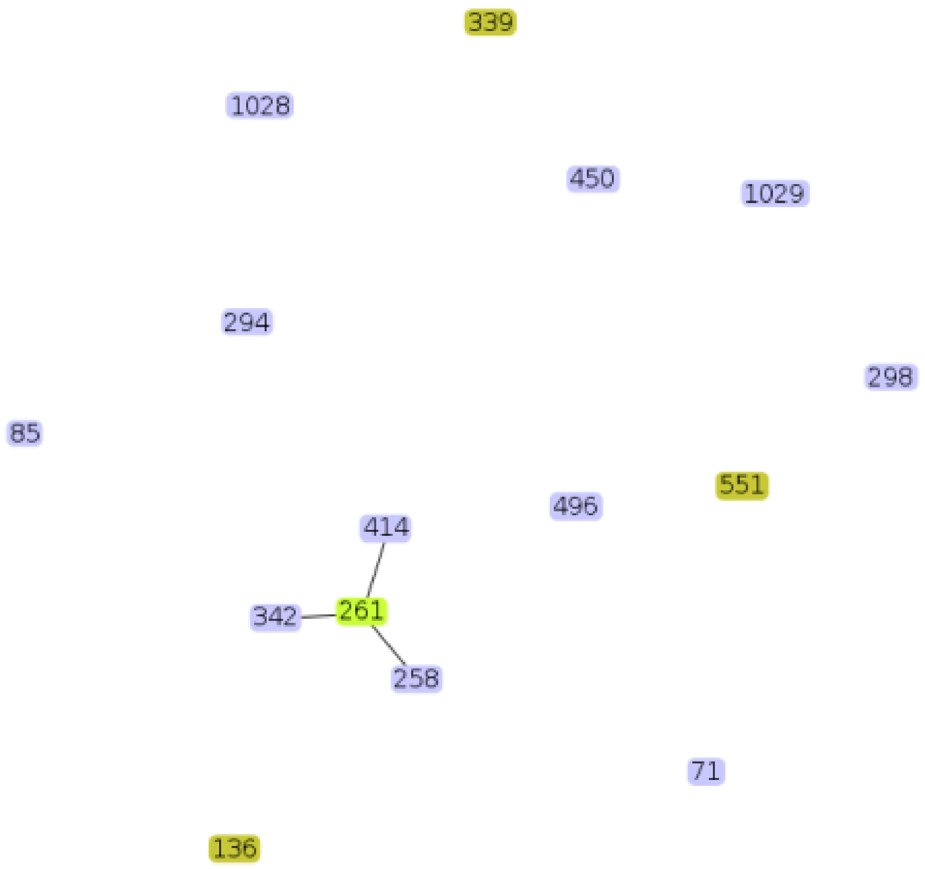

Figure S2:

Clonal Complexes (CCs) distribution for the 2015-2016 collection. Group founders are shown in light green and subgroup founders in dark green.
